# Proceedings of the Twelfth Annual UT-ORNL-KBRIN Bioinformatics Summit 2013

**DOI:** 10.1186/1471-2105-14-S17-A1

**Published:** 2013-10-22

**Authors:** Eric C Rouchka, Robert M Flight

**Affiliations:** 1 Department of Computer Engineering and Computer Science, University of Louisville, Duthie Center for Engineering, Louisville, KY 40292, USA; 2 Department of Chemistry, University of Louisville, Louisville, KY 40292, USA

## 

The University of Tennessee (UT), the Oak Ridge National Laboratory (ORNL), and the Kentucky Biomedical Research Infrastructure Network (KBRIN), have collaborated over the past twelve years to share research and educational expertise in bioinformatics. One result of this collaboration is the joint sponsorship of an annual regional summit to bring together researchers, educators and students who are interested in bioinformatics from a variety of research and educational institutions. This summit provides unique opportunities for collaboration and forging links between members of the various institutions. This year, the Twelfth Annual UT-ORNL-KBRIN Bioinformatics Summit was held at Paris Landing State Park in Buchanan, TN from March 22-24, 2013. A total of 182 participants pre-registered for the summit, with 116 from various Tennessee institutions and 54 from various Kentucky institutions. A number of additional participants came from universities and research institutions from other states and countries, e.g. University of British Columbia, University of Arkansas Medical Sciences, Michigan State University, University of Cincinnati, Iowa State University, etc. Sixty-six registrants were faculty, with an additional 46 students, 43 staff, and 92 postdoctoral participants.

The conference program consisted of three days of presentations. The first afternoon consisted of two workshops, one for Next-Generation Sequence Analysis, and a second on analysis of data resulting from the Conditions Affecting Neurocognitive Development and Learning in Early childhood (CANDLE) project. The remainder was dedicated to scientific presentations divided into three plenary sessions on Next-Generation Sequencing, Translational Bioinformatics, and Systems Biology. In addition, ten short talks were selected from 43 submitted poster abstracts.

## Friday workshops

Ramin Homayouni (University of Memphis) and Zhongming Zhao (Vanderbilt University) opened the Bioinformatics Summit with a workshop titled “Tools and Applications for Next Gen Sequencing.” Michael Dickens from the University of Memphis began the workshop with an overview of bioinformatics tools used in the assembly and annotation of *de novo* genomes. This presentation covered various tools involved in the pipeline aspects of sequencing and quality control, *de novo* assembly, genome annotation, and manual annotation. This included a discussion of WebApollo [http://genomearchitect.org], a web-based community annotation integrated with JBrowse [1]. Pelin Jia from Vanderbilt University followed with a discussion of a pipeline for variant calling within NGS data. She discussed various aspects of the pipeline, including quality control using FastQC [http://www.bioinformatics.babraham.ac.uk/projects/fastqc/]; sequence mapping; post-processing in terms of alignment, marking duplicates, realignment, and base recalibration; variant calling; variant filtering; and variant annotation.

The second half of the opening workshop consisted of workshops on two NGS tools. The first of these tools was MuTect [2] discussed by Huy Vuong from Vanderbilt University. MuTect is a tool used for detection of somatic mutations in cancer which incorporates information from the Catalog of Somatic Mutations in Cancer (COSMIC) [3] as well as tools for calling single nucleotide variants (SNVs). The second tool covered by Qingguo Wang was VirusFinder [4], a tool for detecting viruses and their integration sites using next-generation sequencing data.

Celeste Luketic from Life Technologies closed the first workshop with a discussion of the Ion Torrent^TM^ PGM^TM^ and Proton^TM^ sequencers. These sequencers are based on a semiconductor platform that detects nucleotide incorporation by measuring the resultant change in pH [5]. In addition to discussing the technology and the associated software, Celeste discussed applications using Ion based technologies, including the Ion AmpliSeq^TM^ panels. These ready-to-use panels contain a set of targeted regions for specific diseases, including the cancer hotspot panel, comprehensive cancer panel, inherited disease panel, and sample ID panel for SNP genotyping.

The second workshop of the afternoon focused on “Availability and Uses of CANDLE Genomic Data.” Fran Tylavsky from the University of Tennessee Health Science Center (UTHSC) kicked off the second workshop by giving an overview of a project studying the conditions affecting neurocognitive development and learning in early childhood (CANDLE) [6]. This project, which involves a total of 1474 children (1404 which are active), collected various data using 54 instruments at 24 different time-points, resulting in over 14 million pieces of data, including approximately 900,000 sequence variants at 27,000 sites. Building upon the vast amount of data available through CANDLE, Beni Mozhui from UTHSC followed with a discussion of multiscalar analysis of CANDLE using GeneNetwork [7] and PLINK [8]. GeneNetwork is a platform designed to facilitate genetic studies and integrative systems genetics and models through data storage and data analysis while PLINK is a toolset allowing for whole-genome association and population-based linkage analyses. Rob Williams (UTHSC) closed the second workshop with a hands on demonstration of GeneNetwork functionality by applying it to the CANDLE dataset.

## Session I: next generation sequencing

Jinghui Zhang (St. Jude’s Children’s Research Hospital) began the formal program with a talk titled “Analysis of next-generation sequencing data for pediatric cancer genomes: discoveries, challenges and lessons learned.” In this presentation, Dr. Zhang presented a summary of discoveries resulting from the Pediatric Cancer Genome Project (PCGP), a $65 Million collaboration between St. Jude’s and Washington University in St. Louis [9]. As part of this project, paired next generation sequencing is being performed at the whole genome for both tumor and normal cells for each of 600 pediatric cancer patients. Dr. Zhang discussed a number of interesting discoveries, as well as the development of tools for understanding structural variations in cancer, such as CREST [10].

Tony Capra (Vanderbilt University) followed with a talk titled “Integrating genome-scale data to predict the effect of human-specific non-coding mutations.” Dr. Capra presented a summary of the evolutionary analysis between humans and our closest relatives, chimpanzees. Whole genome sequencing of the chimp genome [11] shows that humans and chimps share a 99% similarity in non-coding regions, with nearly identical protein coding sequences. However, a number of regions have been found where the sequence is much more divergent in humans than between chimpanzees and other distantly related species. These regions, called human accelerated regions (HARs) [12,13] were further studied. A total of 728 HARs are found across the human genome, with 69% in intergenic regions, 21% in introns, 6% in UTRs, and 4% in protein coding regions [14]. HARs tend to be enriched near transcription factors, developmental genes, and genes implicated in diseases. The thought is that these regions function as transcriptional enhancers. Dr. Capra and his group have been working on developing machine learning approaches for detecting possible roles as tissue-specific enhancers of the HARs, and experimentally validating the results.

## Session II: translational bioinformatics

Saturday morning began with a presentation titled “Channotyping Epilepsy – Complexity in Ion Channel Gene Profiles and Personal Risk Prediction”, by Dr. Tara Klassen (Baylor College of Medicine). Dr. Klassen opened the talk by taking the vantage point that perhaps ion channels are the best markers for disease. Over 40 genetic disorders, or channelopathies, caused by ion channels have been characterized, including spinocerebellar ataxia type 13 [15], long and short QT syndrome [16], cystic fibrosis [17], retinitis pigmentosa [18], and several forms of epilepsy. Epilepsy is a spectrum of disorders, affecting 2.2 million people in the United States and 65 Million worldwide [19]. Since the same ion channel gene can cause different excitability disorders in different tissues, a cohort was studied at the Baylor College of Medicine hospitals, including 152 patients with idiopathic epilepsy and 139 with neurologically normal controls. A total of 237 ion channel genes were sequenced and analyzed [20]. From the exploration of the SNPs in these ion channels, it was observed that rare severe ion channel SNPs do not predict epilepsy. In fact, individuals, both with and without epilepsy, can carry multiple mutations in human epilepsy (hEP) genes. Dr. Klassen discussed that it was more of a complex combination of SNPs within hEP genes, and that a systems approach combining sequencing, transcriptomics, proteomics, and modelling was the best approach to understanding the role of ion channel SNPs in epilepsy.

Following Dr. Klassen’s talk was a presentation by Stephen Wong (Weill Cornell Medical College) on “Systems and Chemical Biology Strategies for Drug Repositioning.” In this talk, Dr. Wang discussed approaches to repositioning old drugs, given that the current cost to bring a drug to market costs $1 billion and takes 15 years [21]. Due to this prohibitive cost, the pharmaceutical industry has moved to drug repurposing, resulting in over 40 repositioned drugs [22]. Using computational methodologies, Dr. Wang proposed using knowledge-based, network-based, and disease similarity-based methodologies to aid in drug repositioning. The result is DrugMap Central, which provides an integrative view of multi-dimensional drug data, including basic chemical information, targets, target-related signalling pathways, clinical trial information, and FDA approval information [23].

## Session III: systems biology

Joerg Gsponer (University of British Columbia) began the Sunday sessions with a talk titled “New insights into neurodegeneration by computational approaches.” In this presentation, Dr. Gsponer discussed the role that intrinsically unstructured proteins (IUPs) play in neurodgeneration. Up to one-third of all proteins contain large IUP regions that lack a unique structure [24]. In addition to their lack of higher order structure, many IUPs are also found to form protein aggregates [25]. Cellular systems balance the detrimental and beneficial effect of protein aggregation. IUPs are typically found in low abundance and are short lived. A number of IUPs have been shown to have roles in neurodegenerative diseases such as Alzheimer’s and Parkinson’s diseases. Dr. Gsponer discussed computational approaches to understanding the regulation of aggregation prone proteins, based on the complexity of the 5’ untranslated region (UTR). The 5’ UTR of IUPs typically contains RNA binding motifs, with one example being KHD1 which binds two-thirds of all poly-Q/N proteins [26]. Dr. Gsponer discussed the NeuroGeM Knowledgebase [http://www.chibi.ubc.ca/neurogem/] which contains 1,218 modifiers from 8 disease models, including Alzheimer’s, Huntington’s, poly-q, Parkinson’s, Spinocerebellar ataxia, and amyotrophic lateral sclerosis. He discussed Meta-analysis techniques his group is employing to integrate functional enrichment, disease-specific modifiers, and highly interconnected modifiers to predict modifiers of IUPs for further experimental validation.

Paul Pavlidis (University of British Columbia) closed out the invited speaker portion of the 2013 Summit with a presentation “From gene lists, networks and annotations to function.” The purpose of the presented work was to present the lack of available resources for linking together the genetic basis for diseases and phenotypes. Dr. Pavlidis described Neurocarta, a knowledgebase containing 7,000 genes and 2,000 phenotypes along with supporting evidence linking the genes and phenotypes [27]. Neurocarta was initially developed as a neuroscience resource, and therefore has detailed information about neurodevelopmental disorders. In addition, Dr. Pavlidis discussed issues with annotation resources, pointing to biases and redundancy in Gene Ontology annotations [28] along with the incorrect assumption of “guilt by association” genes which assumes that interacting genes are likely to share similar functions [29,30].

## Posters and short talks

The poster session was held on day two. Forty-three posters were on display, from a variety of different research areas. A number of posters were also selected for short talks. These included “Making data accessible to biologists: small group assignment of correlated genes” (Antony Athippozhy, University of Kentucky); “Giving raw data a chance to talk: a demonstration of de-identified Pediatric Research Database (PRD) and exploratory analysis techniques for possible research cohort discovery and identifiable high risk factors for readmission” (Teeradache Viangteeravat, Children’s Foundation Research Institute); “Power and sample size of two-stage extreme phenotype sequencing design for next generation sequencing studies” (Guolian Kang; St. Jude’s Children’s Research Hospital), “Diffsplice: the genome-wide detection of differential splicing events with RNA-seq” (Yin Hu, University of Kentucky); “A client-oriented workshop on the essentials of next gen sequencing data acquisition and bioinformatics analysis” (Pat Calie, Eastern Kentucky University); “Gene networks in the *Phytophthora capsisci*/*Solanum lycopersicum* pathosystem” (Jordan Bird, University of Tennessee -- Knoxville); “Our strategy to achieve and document reproducible computing” (Nisrine Enyinda, St. Jude’s Children’s Research Hospital); “Isoform reconstruction through molecule inference with statistical isoform selection” (Yan Huang, University of Kentucky); “Using partially ordered sets to represent and predict true patterns of gene response to treatments” (Nam Vo, University of Memphis); and “Query based sampling and multi-layered semantic analysis to find robust network of association between drugs and disease” (Karthikka Ramani Muthukuri, University of Memphis). For full author lists and abstracts see the rest of the supplement.

## Future plans

The 2014 Bioinformatics summit will return to the state of Kentucky and is scheduled for April 11-13, 2014 at Lake Barkley State Park. Potential focus areas include current technological trends in molecular biology, applications of next-generation sequencing, and systems biology.
